# Special Issue “Signal Transductions in Fungi”

**DOI:** 10.3390/jof8050528

**Published:** 2022-05-20

**Authors:** Ulrich Kück

**Affiliations:** Allgemeine & Molekulare Botanik, Ruhr-University, 44797 Bochum, Germany; ulrich.kueck@rub.de

In all living organisms, extracellular signals are translated into specific responses through signal transduction processes. Research with fungal experimental systems has contributed substantially to uncovering the components participating in these processes, such as signaling molecules, receptors, kinases, phosphatases and second messengers. Different signal transduction pathways interact at various cellular levels and form networks that enable the integration of multiple signals for coordinated cellular responses. Such responses include changes in gene transcription, RNA translation and modification, and protein post-translational and conformational states. Most importantly, these processes are dependent on their cellular locations. These molecular events trigger a variety of fungal cellular reactions and developmental processes, including DNA damage response, cell cycle arrest and progression, pathogenic and symbiotic interactions, asexual and sexual propagation, primary and secondary metabolism, stress adaptation, autophagy, and apoptosis.

This special issue includes different research papers and reviews that study the role of signaling molecules, G proteins and receptors, signaling pathways, and multi-subunit complexes allowing the interaction between different signaling pathways, and thus connect upstream regulators and downstream targets. These contributions will promote our understanding of the molecular mechanisms controlling both development and cellular processes in diverse fungal systems [1].

Although there is unambiguous evidence that signaling molecules play an essential role in a broad variety of fungal cellular pathways, less is known about the identity of those regulating fungal development. Eight articles contribute to our current knowledge about the molecular players that participate in initiating cellular differentiation. Abdalmegeed et al. [2] provide results that clearly demonstrate that nitric reductase-dependent nitric oxide levels are involved in hydroxygen-alleviated cadmium toxicity in the basidiomycete *Ganoderma lucidum*. They suggest that rebuilding redox homeostasis increases cysteine and proline levels, thus reducing cadmium accumulation. Inositol phosphate signaling is the subject of an article by Murry and coworkers [3]. Studying the up-regulation of inositol monophosphatase during sexual development in another basidiomycete, *Schizophyllum commune*, they found that altered inositol signaling is involved in tolerance towards metals, such as cesium and zinc, and increased tolerance towards cadmium, which is associated with induced expression of kinases and repression of phosphatases within the inositol cycle. These authors provide a model of how inositol signaling is governed by Ras, G-protein-coupled receptors, and cAMP, and elucidate their different roles in development.

Four articles investigated the effect of osmotic stress on sensing. The relationship between external osmotic stress sensing and the fungicidal activity of phenylpyrrole fludioxonil was the subject of a study by Bersching and Jacob [4]. Essential to both processes seems to be a group III two-component hybrid histidine kinase. However, deletion of the corresponding gene in the plant pathogenic fungus *Magnaporthe oryzae* showed that the molecular mechanisms of fludioxonil action and the perception of osmotic stress are different. In their study, Herrero-de-Dios and co-workers [5] showed that mitogen-activated protein kinase Hog1 is the main kinase responding to osmotic stress in the opportunistic pathogen, *Candida albicans*. In addition to this, a Hog1 mutant strain showed that Hog1 is involved in lipid homeostasis. The authors propose that lipid metabolism contributes to the sensitivity to osmotic stress.

In filamentous fungi, the high-osmolarity glycerol (HOG) signaling pathway is present as a multistep phospho-relay system. Bühring et al. [6] investigated the phosphotransferase system in the rice blast fungus *Magnaporthe oryzae* and addressed the question of how a single gene for a phosphotransfer protein may generate several isoforms to contribute to a multistep phospho-relay system. Their study indicates that alternative splicing is a promising mechanism to generate signal diversity in multistep phospho-relay systems. The HOG pathway is also the subject of a study by Plemenitaš [7]. Changes due to a hypersaline environment were investigated by comparing the halotolerant black yeast *Hortaea werneckii* and the obligate halophilic fungus *Wallemia ichthyophaga* with the salt-sensitive yeast *S. cerevisiae*. The author found important structural differences in the HOG pathway components. In conclusion, novel mechanisms of adaptation were driven by changes in salt concentration. 

Reactive oxygen species (ROS), such as H_2_O_2,_ are involved in the signaling of all living organisms and are implicated in a variety of biological processes [8]. Carrasco-Navarro and Aguirre [9] used a global phosphoproteome analysis to investigate the effect of H_2_O_2_ in the ascomycete *Aspergillus nidulans*. They found that H_2_O_2_ affects the phosphorylation of critical regulatory nodes of phosphoinositide, MAPK and TOR signaling, as well as the phosphorylation of multiple proteins involved in diverse developmental processes.

Signaling molecules play a central role in the interspecies or even interkingdom communication of all living organisms. Kwon et al. [10] are interested in the interaction between the fungal pathogen *Ustilago maydis* and its plant host maize. They investigated extracellular vesicle-associated mRNAs secreted by the maize smut pathogen. Using isolated vesicles, they show by RNA-seq analysis that several mRNAs are enriched and upregulated during infection, and may thus participate in the interaction between the pathogen and host plant.

Two articles provide quite different experimental systems to understand signal transduction pathways. Groth, Schunke and coworkers [11] used a fluorescent microscopy approach to reveal an actin-related protein as a new marker protein to track active polar hyphal growth. Monitoring this protein allows one to study growth dynamics and ascospore germination, and the interpretation of chemotropic growth processes, thus enabling the study of polar growth processes in living fungal cells. Seike and co-workers [12] present an ecological study with different wild type strains from the yeast *Schizosaccharomyces japonicus*. Interestingly, strains isolated from diverse host *Drosophila* species showed a high frequency of sexual sporulation, even under nitrogen-abundant conditions. The authors suggest that their ecological and evolutionary study provides a basis for understanding the sporulation mechanism.

Classical paradigms show that G-protein-coupled receptors (GPCRs) transduce signaling through G proteins. For example, they act as molecular switches in the transduction of intracellular signaling [13]. Since their initial discovery, several variant types of receptors have been described contributing to a broad variety of signaling pathways. Several contributions in this special issue deal with G-proteins, receptors, transmembrane ion channels, kinases and phosphotransferases, and most of them are non-conventional. Two articles deal with the β subunits of G-proteins. Lim et al. [14] functionally characterized a deletion strain from the fungal pathogen *Aspergillus fumigatus*, lacking a gene for a Gβ-like protein. They showed by organ-specific transcriptional analysis that the Gβ-like protein is essential for completing sexual development. Further roles are associated with phagocytosis by alveolar macrophages, biosynthesis of the cell wall, and oxidative stress responses. A similar experimental approach was used by Tang and coworkers [15], on the necrotrophic phytopathogenic fungus *Botrytis cinerea*, which causes gray mold disease in crops. A deletion strain lacking the G protein β subunit gene shows significantly reduced transcription of genes involved in cAMP signaling. From their results the authors conclude that the Gβ protein controls development and virulence through both the cAMP and MAPK signaling pathways.

Transient receptor potential (TRP) proteins are thought to constitute transmembrane ion channels with highly diverse permeation and gating properties. Wang et al. [16] characterize a putative TRP protein in the fungus *Aspergillus nidulans*. Gene knock-out experiments and fluorescence microscopy data provide evidence for a Golgi-localized TRP-like protein that performs an important function in conidiation, cell wall integration and cellular calcium equilibrium.

Pheromone receptor genes are involved in sexual mating of the tetrapolar basidiomycete *Schizophyllum commune*, but the function of other pheromone receptor-like genes has not yet been described. Wirth et al. [17] characterize four B-receptor-like genes, which are orthologs of the *S. cerevisiae* Ste3a-factor receptor. The data from transcriptome expression analysis and overexpression experiments provide evidence that these genes play a functional role in vegetative growth, possibly by determining growth direction.

Signaling pathways activate or silence DNA sequence-specific regulators, such as transcription factors (TFs), which have been intensively studied in members of the Saccharomycotina. In this context, Extebeste [18] provides a bioinformatic study of genes predicted to encode TFs in a member of the Pezizomycotina, namely *Aspergillus nidulans*. Analyses of the expansion of different families of TFs indicate that the duplication of TFs has an impact at the species level, and the author concludes that the expansion of Zn_2_Cys_6_ TFs is mainly due to dispersed duplication events.

The initial characterization of diverse signaling pathways considered them as linear pathways that do not interact with each other. However, depending on certain physiological conditions, these pathways may enable crosstalk between each other, probably to increase the range of specific cellular responses without increasing the number of components constituting a pathway. The following articles investigated the interplay between two signaling cascades in diverse fungal systems by comparing the phenotypes of signaling-component mutants.

Starke et al. [19] investigated signaling cascades in the plant pathogen *Verticillium dahlia*. They constructed mutants related to the pheromone response MAPK pathway, or the endoplasmic reticulum-associated unfolded protein response (UPR), both affecting fungal growth, resting structure development, and virulence of the pathogen. The similar phenotypes of signaling-component mutants implied that an interplay exists between UPR and pheromone response MAPK signaling. Similarly, the interplay between two signaling pathways was studied by Moreno-Ruiz et al. [20] They investigated the stress-activated protein kinase signaling (SAPK) and mitogen-activated protein kinase (MAPK) signaling network in *Trichoderma atroviride*, a mycoparasitic fungus. The comparative analysis of mutants lacking genes for components of the above-mentioned signaling pathways showed that they are essential for establishing fully functional mycoparasitism.

The integration of components of several pathways is facilitated by multi-subunit complexes that allow crosstalk between upstream and downstream effectors of the complex [21]. A well-characterized multi-subunit complex is the striatin-interacting phosphatases and kinases (STRIPAK) complex [22], for which a recently identified coiled-coil protein SCI1 was demonstrated to co-localize around the nucleus. Groth, Schmitt and co-workers [23] performed pulldown experiments with SCI, identifying a transmembrane nucleoporin as a potential nuclear anchor of STRIPAK. The authors speculate that this nucleoporin may temporarily anchor STRIPAK to the nuclear envelope. The impact of STRIPAK on diverse fungal developmental processes is summarized by Kück and Stein [24]. Furthermore, the crosstalk of STRIPAK through controlling the dephosphorylation of subunits from other signaling complexes such as the septation initiation network (SIN) is discussed. Finally, the article provides a perspective on how so far non-characterized proteins may function as receptors connecting mitophagy to the STRIPAK signaling complex.

Mitophagy, the selective vacuolar/lysosomal degradation of mitochondria, is also involved in the aging process, basically occurring in all living beings. The molecular networks that affect this biological process are associated with mitochondria and pathways involved in the biogenesis and quality control of these organelles [25]. The review by Osiewacz and Schürmanns [26] serves as an excellent summary of different signaling pathways implicated in the control of the aging process in the model fungus *Podospora anserina*. For example, the stability of mitochondrial DNA affects lifespan, as does the mitochondrial level of reactive oxygen species (ROS), which has to be balanced since ROS, at low abundance, are essential signaling molecules, but at higher levels cause damage to all kinds of biomolecules. However, when the abundance of ROS passes critical limits, different pathways are induced to cope with the resulting impairments. One of these processes is the degradation of damaged, or excess mitochondria via mitophagy.

Improving our understanding of signal transduction networks will require scientific work by many investigators, and I hope that this special issue contributes to this effort. I very much appreciate receiving the articles and reviews from each of the authors, making this special issue possible. Furthermore, I am grateful to the “Journal of Fungi” to have this opportunity to gather articles from the fungal community presenting remarkable work that advances our understanding of “Signal Transductions in Fungi”.
